# Pregnancy Intention: A Concept Analysis

**DOI:** 10.3390/nursrep16100351

**Published:** 2026-09-30

**Authors:** Merilyn Guerra-Ramírez, María Elisa Moreno-Fergusson

**Affiliations:** Faculty of Life Sciences and Well-Being, Universidad de La Sabana, Chía 250001, Colombia; cuidamas@unisabana.edu.co

**Keywords:** reproductive decision-making, pregnancy planning, preconception, reproductive health, concept analysis, nursing

## Abstract

Pregnancy intention is widely used in sexual and reproductive health research and practice, yet its conceptual boundaries remain insufficiently defined. This imprecision limits consistent interpretation and shapes how nurses and other health professionals assess and support women’s reproductive decision-making across the reproductive continuum. **Objective**: to clarify the concept of pregnancy intention and to propose a conceptual definition applicable to nursing practice, sexual and reproductive health care, and health promotion. **Methods**: A concept analysis was conducted using Walker and Avant’s eight-step method. A structured search of the literature published between 2014 and 2025 in English, Spanish, and Portuguese was carried out in PubMed, Web of Science, SAGE, ScienceDirect, SciELO, and Dimensions, complemented by lexicographic sources and grey literature. Data were extracted and analyzed to identify the concept’s uses, defining attributes, antecedents, consequences, and empirical referents. **Results:** Pregnancy intention emerged as a multidimensional concept defined by three attributes: temporal orientation toward a future pregnancy, conscious deliberation about conceiving, and contextual appraisal of readiness. It is defined as a woman’s conscious and prospective disposition toward a future pregnancy, involving deliberation about conceiving and contextual appraisal of her readiness in relation to the personal, relational, socioeconomic, and health-related circumstances she considers relevant to the decision. **Conclusions**: The proposed definition provides a theoretically coherent basis for reproductive assessment, preconception care, and woman-centered health promotion. By recognizing the personal, social, and structural conditions relevant to women’s health, including socioeconomic conditions that may contribute to broader social determinants of health, it also supports a health-equity perspective and provides a basis for future prospective measurement that reflects women’s reproductive experiences.

## 1. Introduction

Women’s health is a global priority and an essential component of the social, economic, and cultural development of communities, recognizing women as agents of change who contribute to the well-being of their families and of society [1]. In this context, sexual and reproductive health promotes women’s right to decide whether to have children, when to do so, and under what circumstances, in keeping with their life projects and reproductive preferences [1,2].

Over the past five decades, social and demographic changes have profoundly reshaped women’s reproductive trajectories. Broader access to higher education, women’s growing participation in the labor market, and greater autonomy in decision-making have redefined the timing and circumstances in which motherhood is considered [3,4,5,6,7]. As a result, the decision to have children no longer responds solely to biological or familial factors but has become a deliberative process influenced by personal, economic, relational, and sociocultural conditions whose importance and meaning vary across women and contexts.

These reproductive decisions are not made under equivalent social and structural conditions. Socioeconomic disadvantage, gendered power relations, partner violence, and differences in access to health and reproductive health services may shape the circumstances in which women formulate and act on their pregnancy intentions, with differences also observed across social and racial/ethnic groups [8,9,10,11]. From a health equity perspective, conceptualizing pregnancy intention solely as an individual preference may obscure the social and structural conditions that shape women’s opportunities to consider, plan, and prepare for a future pregnancy, as well as their access to appropriate health care and support. A clearer conceptualization should therefore recognize that pregnancy intention is situated within social and structural circumstances that may influence both the process of pregnancy-related decision-making and subsequent health trajectories.

Against this backdrop, pregnancy intention has attracted growing interest in sexual and reproductive health research and practice. The concept has been used across disciplines such as obstetrics and gynecology, midwifery, women’s health nursing, public health, and community care, where it is linked to reproductive decision-making, preconception care, and the planning of interventions for women of reproductive age [8,12,13,14,15]. However, its use remains heterogeneous and lacks a widely accepted conceptual delimitation. In the literature, pregnancy intention is often used interchangeably with related concepts such as pregnancy planning, preconception, pregnancy desire, and reproductive decision-making, which produces conceptual ambiguity, hampers comparison across studies, and limits the development of care strategies grounded in precise conceptual language [16].

Although pregnancy planning and preconception have been the subject of previous concept analyses [17,18], these concepts represent related but conceptually distinct phenomena. Pregnancy planning describes an action-oriented process comprising the behaviors and preparations undertaken to achieve a pregnancy, whereas preconception refers to the period before pregnancy and to the set of interventions aimed at improving health before conception [12]. By contrast, pregnancy intention represents the cognitive, deliberative, and affective disposition toward a future pregnancy, which may precede, accompany, or even exist independently of planning behaviors. Consequently, a woman may intend to become pregnant without having initiated concrete steps toward it, just as she may engage in planning behaviors with a weak, shifting, or ambivalent intention. Distinguishing these concepts is essential to avoid their interchangeable use in research and to support a more precise assessment of women’s reproductive experiences. Recent literature has considered pregnancy intention in relation to ambivalence, prospective and retrospective assessment, the use of intendedness categories, and reproductive autonomy [19,20]. Studies have described mixed or changing feelings toward a future pregnancy [21], differences between prospective and retrospective reports of pregnancy intention [22], and the use of intendedness categories to describe and classify pregnancy experiences [23]. Other studies have examined pregnancy intention in relation to women’s reproductive autonomy and decision-making within their social and cultural contexts [19,20]. Taken together, these perspectives highlight both conceptual ambiguity and questions regarding how pregnancy intention has been assessed and classified; the present analysis primarily addresses the former by clarifying its defining characteristics.

Since Fischer and colleagues highlighted the conceptual ambiguity surrounding the terms intended, planned, and wanted pregnancy [16], subsequent literature has continued to question the conceptualization and measurement of pregnancy intendedness. Although analyses of related concepts have helped clarify specific dimensions of the reproductive process, pregnancy intention itself has not been systematically examined in terms of its defining attributes, antecedents, consequences, and empirical referents. This ongoing conceptual ambiguity constrains the theoretical development of the concept, hampers comparison across studies, and limits the development of assessment instruments and nursing interventions grounded in precise conceptual language.

The present study therefore aims to analyze the concept of pregnancy intention using Walker and Avant’s methodology [24] in order to identify its uses, defining attributes, antecedents, consequences, and empirical referents, and to propose a conceptual definition that strengthens research, clinical practice, and the conceptual development of nursing in the field of sexual and reproductive health.

## 2. Materials and Methods

A concept analysis was conducted following the eight-step methodology proposed by Walker and Avant [24], comprising: (1) selecting the concept, (2) determining the purpose of the analysis, (3) identifying its uses, (4) determining the defining attributes, (5) constructing a model case, (6) constructing additional cases, including borderline, related, and contrary cases, (7) identifying antecedents and consequences, and (8) defining the empirical referents. Although presented as a sequence of methodological steps, the analysis was conducted iteratively. Defining attributes, cases, antecedents, consequences, and empirical referents were reviewed and refined in relation to one another throughout the analytic process to clarify the conceptual boundaries of pregnancy intention.

Walker and Avant’s approach was selected because it supports the identification of defining attributes, antecedents, consequences, and empirical referents and the formulation of an operational definition. Rather than examining the historical evolution of the concept, the analysis focused on its current definitions and uses across disciplinary and contextual sources. Accordingly, no critical risk-of-bias appraisal was performed, as the purpose of a concept analysis is to examine how a phenomenon has been described, defined, and used across different contexts [24]. Empirical studies, conceptual publications, dictionaries, and grey literature were therefore considered when they contributed relevant information to the conceptual analysis.

For Step 6, model, borderline, related, and contrary cases were constructed because these cases were considered relevant to delineating the conceptual boundaries of pregnancy intention. Invented and illegitimate cases were not constructed because they were not considered necessary to further clarify the concept within the scope of this analysis. The analysis was delimited to the woman’s own pregnancy intention, understood as her disposition toward a future pregnancy. Accordingly, publications concerning surrogacy, gestational surrogacy, or reproductive decisions regarding another person’s pregnancy were excluded because they correspond to conceptually distinct phenomena outside the purpose of this study.

The literature search was conducted in PubMed, Web of Science, SAGE Journals, ScienceDirect, SciELO, and Dimensions, complemented by specialized dictionaries and grey literature. The database-specific search strategies were developed around the terms “pregnancy intention”, “pregnancy intentions”, and “intended pregnancy”, combined with Boolean operators and conceptual terms related to definition, meaning, conceptualization, measurement, and concept analysis. The search was intentionally centered on terms naming the focal concept; related concepts such as pregnancy planning, preconception, and pregnancy desire were examined analytically through the retrieved literature rather than searched as separate terms to keep the concept analysis bounded to pregnancy intention. PRISMA 2020 was used solely as a reporting aid to improve transparency in the literature search and study-selection process [25]. The complete database-specific search strategies, search fields, filters, and search dates are provided in Supplementary Material S1. Search-level exclusions were applied through NOT operators for terms related to adolescence and surrogacy, whereas eligibility exclusions for studies focused exclusively on infertility treatment or pre-existing morbid conditions were applied during screening.

Publications in English, Spanish, and Portuguese published between 2014 and 2025 were included when they addressed pregnancy intention in women of reproductive age and provided information relevant to its conceptualization, measurement, associated factors, consequences, or relevance to nursing and reproductive health. The search period was established to capture contemporary literature while retaining foundational publications when they made a direct contribution to the conceptual development of pregnancy intention. Accordingly, the work by Bloch et al. [13] was retained because of its direct conceptual relevance despite being published before the predefined search period. Studies focused exclusively on adolescent pregnancy, infertility treatment, or pre-existing morbid conditions that did not address pregnancy intention as a primary or secondary concept were excluded. Information from the included sources was organized in a structured extraction matrix recording author/source, year, country or setting, language, publication type, uses of the concept, defining attributes, antecedents, consequences, and empirical referents, consistent with the analytic components proposed by Walker and Avant. Both authors independently reviewed the extracted information and resolved discrepancies by consensus.

To reduce subjectivity in the analytic process, predefined criteria were applied to distinguish defining attributes from antecedents, consequences, contextual circumstances, and empirical referents. Terms, definitions, and descriptions related to pregnancy intention were compared iteratively across the included sources to identify recurring conceptual elements. An element was considered a defining attribute when it was described as an essential characteristic of pregnancy intention, contributed to distinguishing the concept from related concepts, and represented a characteristic of the concept itself rather than a preceding condition, an outcome, or an observable indicator. Elements referring primarily to contextual circumstances, specific measurement procedures, or behavioral outcomes were not classified as defining attributes. Antecedents, consequences, and empirical referents were identified according to their respective definitions within Walker and Avant’s approach and were kept conceptually distinct from the defining attributes [24]. Each candidate attribute was examined through model, borderline, and contrary cases to assess its defining status within pregnancy intention. These criteria were systematically applied across the literature to distinguish defining attributes, antecedents, consequences, and empirical referents. Both authors reviewed the resulting analysis, which informed the illustrative cases and the final conceptual definition.

## 3. Results

The results are presented following the methodological sequence proposed by Walker and Avant [24]. First, the characteristics of the included sources are described to contextualize the evidence that underpinned the conceptual analysis.

### 3.1. Characteristics of the Included Sources

The literature search identified 78 records in the databases consulted. After removing six duplicate records, 72 titles and abstracts were screened, of which 16 were excluded for not meeting the eligibility criteria. Subsequently, 56 full texts were reviewed, and 36 publications were excluded for not addressing pregnancy intention as a primary concept or for falling outside the scope defined for this analysis. Finally, 20 studies were included through the systematic search; to these, 3 grey-literature sources identified by other methods were added, for a total of 23 sources included in the concept analysis. The selection process is presented in Figure 1.

The included publications comprised quantitative, qualitative, and mixed-methods studies, systematic reviews, and meta-syntheses, conducted mainly in the United States, Australia, Sweden, China, Brazil, Uruguay, Bangladesh, and Denmark. The studies were published in English, Spanish, and Portuguese, reflecting the geographic, methodological, and disciplinary diversity of the evidence analyzed. Consistent with concept-analysis methodology, the analysis was further complemented by grey-literature sources (Gawel [26], Planned Parenthood [27], and United Healthcare [28]), which contributed relevant conceptual elements and, having been identified through other methods, were incorporated into the included sources. The characteristics of the 23 sources analyzed are presented in Table 1.

### 3.2. Selection of the Concept

The literature analysis identified pregnancy intention as a concept used across sexual and reproductive health research and practice, with variability in its definition, application, and operationalization and overlap with related concepts such as pregnancy planning, pregnancy desire, and preconception. These findings supported the selection of pregnancy intention as the concept for analysis and guided the subsequent examination of its defining characteristics.

### 3.3. Purpose of the Analysis

The analysis was undertaken to clarify the meaning and conceptual boundaries of pregnancy intention. Examination of the included sources identified its uses, defining attributes, antecedents, consequences, and empirical referents, which provided the basis for distinguishing pregnancy intention from related concepts and formulating its conceptual definition.

### 3.4. Uses of the Concept

The literature review showed that pregnancy intention is not a standardized descriptor in the main health-sciences thesauri and dictionaries in English, Spanish, or Portuguese. The analysis therefore considered how the term is used across different disciplinary and applied contexts. The Oxford Learner’s Dictionaries defines intention in relation to what a person intends or plans to do, or their aim, whereas pregnancy refers to the period between conception and birth [42]. From a phenomenological perspective, Hedwig [43] discusses intention in relation to the directedness of mental activity and purposive action. In clinical and reproductive-health literature, pregnancy intention is used to describe whether and when a pregnancy is intended and to inform reproductive assessment and care [31,38,41]. In obstetric and midwifery literature, pregnancy intention is used to describe whether and when a pregnancy is intended and to support reproductive counselling and care [31,38,41]. In public health and demographic research, it is used to classify pregnancy intendedness and examine patterns associated with reproductive behaviors and outcomes [10,11,34,37]. In nursing, the term is applied in reproductive health assessment and preconception care [26,27,28,35,36].

In instrument-based research, pregnancy intention is operationalized through questions and measures addressing intendedness, timing, desirability, and related reproductive preferences [39,40]. These uses show that pregnancy intention is applied differently across disciplinary and measurement contexts. The defining attributes of the concept were examined separately in the subsequent stage of the analysis.

### 3.5. Defining Attributes

The analysis of the included sources identified three defining attributes of pregnancy intention: temporal orientation toward a future pregnancy, conscious deliberation about conceiving, and contextual appraisal of readiness. These attributes were identified by comparing the definitions, descriptions, and uses of pregnancy intention across the included sources and applying the predefined analytical criteria described in the Methods. The analysis distinguished characteristics intrinsic to the concept from antecedents, consequences, contextual circumstances, and empirical referents. Together, the three attributes describe pregnancy intention as a prospective and deliberative disposition in which a woman considers her readiness for a future pregnancy in relation to the circumstances relevant to her situation.

Table 2 presents the identified defining attributes and their relevance to nursing practice and sexual and reproductive health care, while Table 3 shows the sources supporting each attribute.

#### 3.5.1. Temporal Orientation Toward a Future Pregnancy

The first defining attribute is temporal orientation toward a future pregnancy. In the sources that addressed the temporal dimension, pregnancy intention was conceptualized in relation to a future pregnancy or its anticipated timing [13,26,27,37,39,41]. This prospective orientation establishes temporality as an inherent dimension of pregnancy intention and supports its recognition as a defining attribute of the concept. This understanding is consistent with the linguistic and phenomenological literature on intention discussed in the uses section [42,43], which conceptualizes intention as inherently directed toward a future state or event. Accordingly, temporal orientation toward a future pregnancy is understood as intrinsic to pregnancy intention. The specific timeframes reported in the literature [26,27,37], ranging from approximately six months to five years, illustrate the variability in how this temporal dimension has been operationalized and measured across studies, rather than indicating a single temporal interval defining the concept. This temporal orientation distinguishes pregnancy intention from other expressions of reproductive desire by establishing an orientation toward a future pregnancy that has not yet occurred.

#### 3.5.2. Conscious Deliberation About Conceiving

The second defining attribute is conscious deliberation about conceiving. Across the included sources, pregnancy intention was described in relation to cognitive and affective processes through which women consider whether they want a future pregnancy and reflect on their readiness to conceive [14,27,28,31,32,33]. This deliberative process may involve evaluating personal preferences, feelings, beliefs, perceived readiness, and the implications of pregnancy for one’s life circumstances. The defining element is the conscious appraisal involved in forming an orientation toward a future pregnancy, rather than any specific action or outcome associated with the reproductive decision. This attribute distinguishes pregnancy intention from pregnancy desire or preference when these are expressed without an accompanying process of conscious consideration regarding the possibility of conceiving.

#### 3.5.3. Contextual Appraisal of Readiness

The third defining attribute is the contextual appraisal of readiness, understood as the woman’s evaluative consideration of the circumstances she regards as relevant to a future pregnancy. This appraisal is a necessary feature of pregnancy intention, since to form an intention, a woman weighs her readiness against her circumstances. What varies is not whether this appraisal occurs, which is always entailed, but which circumstances are weighed and how much importance each carries. Consistently, the literature has identified four sub-dimensions that make up this attribute: health status, support network, socioeconomic conditions, and the social roles women hold. These elements interact dynamically during the deliberative process and help to determine when and under what circumstances a woman considers it appropriate to begin a pregnancy.

Health status comprises a woman’s appraisal of her physical and mental conditions and of how these may influence the possibility and timing of conceiving [34,37]. The support network includes the quality of the partner relationship, family support, beliefs and values, and other significant relationships that may facilitate or hinder the reproductive decision [10,35,40]. Socioeconomic conditions, as one component of the broader social determinants of health, may include aspects such as employment, financial stability, education, and access to health services [11,32,34,37], whereas women’s social roles and responsibilities refer to the family, educational, and occupational circumstances that women may consider in relation to their reproductive plans [8,37,41]. Together, these circumstances illustrate the range of contextual factors that may be appraised when considering readiness for pregnancy. Their relevance is individual and context-dependent, and the importance assigned to each may vary across women and circumstances.

The social-roles dimension was supported by one peer-reviewed source and one grey-literature source [8,37,41]. Although the available evidence remains limited, these sources indicate that educational, occupational, relational, and life-course circumstances may be considered when women evaluate the timing and appropriateness of a future pregnancy. This dimension should therefore be interpreted as a contextual aspect of readiness rather than as a universal or independently established feature of pregnancy intention.

### 3.6. Case Construction

Following Walker and Avant [24], model, borderline, related, and contrary cases are constructed. Each narrative is followed by an analysis of how it relates to the defining attributes. The circumstances described in the model and borderline cases, such as a partner relationship, employment, or financial security, are presented as considerations that each woman weighs, not as requirements of pregnancy intention.

#### 3.6.1. Model Case

Ana, 27, has been in a stable partner relationship with Guillermo for four years and is completing her graduate studies. Together they have agreed to try to conceive within the coming year. Both express positive anticipation and have begun to adopt healthier lifestyles in preparation for pregnancy; by their own appraisal, their financial situation and family support make them feel ready for parenthood.

Ana’s situation exemplifies the three defining attributes: a prospective, temporally specified orientation toward pregnancy; conscious deliberation that integrates emotional and relational readiness; and contextual appraisal of readiness, as she and her partner appraise their circumstances as favorable. These specific conditions—partner relationship, financial stability, and family support—are the considerations that Ana herself weighs; another woman might form an equally clear intention while weighing entirely different conditions. The circumstances a woman can consider, and the options available to her, may be shaped by her social position and by broader social determinants of health [9,11]. Thus, pregnancy intention is formed within social and structural contexts that may influence the conditions women are able to consider when appraising their readiness for pregnancy.

#### 3.6.2. Borderline Case

Patricia, 23, and her partner Rafael, 26, have been married for three years. They actively discuss whether they are ready to have a child and consider their financial situation, employment stability, and mortgage as factors in deciding whether they feel prepared for a pregnancy. However, they have not established any timeframe or anticipated when they would want to become pregnant.

Patricia’s case exhibits two of the three defining attributes. Conscious deliberation about conceiving is present, as she and Rafael actively discuss whether they are ready for a child, and contextual appraisal of readiness is present, as they weigh their financial situation, employment stability, and housing. However, temporal orientation toward a future pregnancy is absent, since they have not established a timeframe or anticipated when they would seek conception. The case is borderline because these two attributes are present while the temporal orientation has not yet formed. Patricia’s deliberation is also shaped by material conditions; for couples with fewer economic resources, similar circumstances may weigh differently in the appraisal of readiness [8,11].

#### 3.6.3. Related Case

Susana, 30, recently had her first child. She and her partner would like to have a second child but want to wait longer. Because she experienced pre-eclampsia in her first pregnancy, Susana is attentive to her health, and the couple deliberately postpones conception while preparing physically and emotionally for a future pregnancy.

Susana’s case is related but distinct. It illustrates a concept adjacent to pregnancy intention, birth spacing, or the management of the interpregnancy interval, in which reproductive desire is present, and a future pregnancy is anticipated, but the intention is deliberately suspended for reasons of health and readiness. It shares the conscious-deliberation and contextual-appraisal dimensions of pregnancy intention and differs in its immediate temporal orientation, directed at postponement rather than at conception within a specified horizon. This case clarifies the boundary of the concept with interpregnancy planning.

#### 3.6.4. Contrary Case

Estela, 39, has never wished to have children, and her partner shares this position. She describes her purpose in terms of personal and professional growth and finds deep fulfillment in her environmental work and in caring for the dog they adopted.

Estela shows no temporal orientation toward a future pregnancy, no conscious deliberation about conceiving, and no contextual appraisal of readiness: a clear illustration of what pregnancy intention is not. The absence of these attributes should not be read as an individual failing; it may reflect social and structural conditions that constrain how readiness is appraised [8,9]. Across these cases, the appraisal of readiness is consistently situated within each woman’s social and structural circumstances, underscoring the relevance of a health-equity perspective for interpreting pregnancy intention.

### 3.7. Antecedents and Consequences

#### 3.7.1. Antecedents

Antecedents are the conditions that must be present for the concept to occur [24]. They are the foundational preconditions without which the defining attributes cannot exist and are therefore distinct from the contextual appraisal of readiness, which is a defining attribute of the concept itself. The first is reproductive capability, that is, being of reproductive age or otherwise having the means to achieve a pregnancy; with assisted reproduction, biological reproductive age is not strictly necessary, and what is required, consistent with the self-referential scope of this analysis, is that a future pregnancy be possible for the woman.

The second is reproductive autonomy and agency, understood as the woman’s own freedom and capacity to form her reproductive decisions and act on them; this refers to personal self-determination and decisional agency, not to the availability or use of any specific reproductive-health service or method, and without a minimum of it, an intention cannot be authentically formed or exercised. The third is awareness of reproduction, a basic understanding of reproduction, fertility, and timing that makes a deliberate orientation toward, or away from, a future pregnancy possible. The fourth is a prospective non-pregnant state, since pregnancy intention is prospective and presupposes that the woman is oriented toward a future pregnancy rather than already carrying the intended one.

The relational context may also be relevant to pregnancy intention. Evidence from Brazil indicates that marital status and having a partner are associated with reproductive intentions, suggesting that relationship circumstances may form part of the context in which women consider pregnancy [32]. For this reason, and to preserve the self-referential scope of the concept—the woman’s own intention rather than a shared or partner-driven decision—the partner relationship is situated among the contextual appraisal of readiness (a defining attribute that the woman herself weighs) and is not treated as a universal precondition. Financial security, stable employment, and effective management of health conditions are likewise part of the contextual appraisal of readiness rather than antecedents and are treated as such to avoid conceptual overlap [14,18,44].

#### 3.7.2. Consequences

Consequences are outcomes that may follow from the occurrence of the concept [24]. They are potential rather than necessary: a woman who intends a pregnancy will not inevitably experience the outcomes that follow, but pregnancy intention increases their likelihood.

As probabilistic outcomes, the analysis identified a greater likelihood of preconception engagement, which includes seeking preconception health assessment and fertility-related care and physical, emotional, and psychological preparation for pregnancy [28]; earlier and more sustained engagement with services, with earlier initiation of and continued attendance at prenatal services and possible associated reductions in maternal-perinatal risk through changes in health behaviors [33]; adjustments in life planning, with possible changes in partner and family dynamics and in financial planning when the woman or the couple reorganizes resources and roles in anticipation of a pregnancy [37]; and affective outcomes, as more positive reproductive experiences are reported when the intention is sustained by favorable contextual conditions, whereas anxiety and stress may follow when those conditions are constrained [38,41].

Pregnancy intention may also keep longer-term outcomes on the radar for preventive purposes. By prompting earlier attention to modifiable preconception risks, it may contribute to reducing the risk of chronic disease in the offspring, a distal association that operates through preventable pathways and does not follow directly from the intention itself [31,45]. Without attention to these contextual conditions, existing disparities in preconception preparation and engagement with care may remain unaddressed [9,10].

### 3.8. Empirical Referents

The empirical referents derived from the defining attributes include the statement of a timeframe for conception, the initiation, discontinuation, or non-initiation of contraception with conception in view, explicit discussion of the reproductive project with a partner, where relevant, initiation of preconception health behaviors or a preconception consultation, and articulation of the conditions considered necessary or desirable for readiness.

These empirical referents have been operationalized through different measurement approaches. Survey questions have addressed the timing and desirability of pregnancy, including the following screening question, translated from Spanish: “Did you want to become pregnant at that time? Later? Not at all?” [33], as well as timing-preference items using different prospective timeframes [13,32,37]. The London Measure of Unplanned Pregnancy (LMUP [46]) is a validated six-item instrument that assesses dimensions including feelings toward pregnancy, discussion with the partner, and preparation for pregnancy. However, the LMUP is designed for retrospective application. Prospective instruments for preconception assessment remain underdeveloped, a gap that limits professionals’ ability to assess pregnancy intentions before they are resolved by an actual pregnancy outcome.

## 4. Proposed Conceptual Definition

Based on the defining attributes identified through the analysis, pregnancy intention is proposed as an operational definition, understood as a woman’s conscious and prospective disposition toward a future pregnancy, involving deliberation about conceiving and contextual appraisal of her readiness in relation to the personal, relational, socioeconomic, and health-related circumstances she considers relevant to that decision.

In Walker and Avant’s approach, the defining attributes provide the basis for formulating the conceptual definition, whereas the empirical referents identified in Step 8 indicate how the concept may be recognized or observed in practice. Accordingly, the definition proposed in this analysis is a conceptual definition: it specifies the defining attributes of pregnancy intention rather than prescribing a specific method of measurement. The empirical referents identified in the analysis may contribute to a more coherent operationalization of pregnancy intention in future research by providing observable elements linked to its defining attributes. Given the current lack of a unified conceptual and measurement approach to pregnancy intention, these referents may also provide a basis for developing and testing more consistent prospective measures. The development and empirical testing of such an approach therefore represents an important next step following this concept analysis.

## 5. Discussion

This concept analysis supports a reframing of how pregnancy intention is understood and applied in reproductive and preconception care. The three defining attributes identified, namely temporal orientation, conscious deliberation, and contextual appraisal of readiness, form an internally coherent conceptual architecture. This architecture reflects the experiential complexity documented in qualitative, quantitative, and mixed-methods sources. At the same time, it remains operationally manageable for clinical assessment and health-promotion design.

A central finding is that the appraisal of contextual circumstances forms part of the defining characteristics of pregnancy intention rather than functioning merely as a moderating factor. The contextual nature of pregnancy intention also has implications for culturally responsive care. The analysis suggests that the importance attributed to the partner relationship may vary across cultural contexts. In some settings, particularly those described in Latin American literature, a stable partner relationship is regarded as an important condition when considering a future pregnancy, whereas the extent to which the partner participates in the reproductive decision may vary across contexts [15,32,39]. This finding highlights the importance of considering the woman’s relational and cultural context when assessing pregnancy intention. For nursing and reproductive health practice, assessment should therefore explore the significance of the partner relationship from the woman’s own perspective and within her social and cultural context, rather than assuming that its meaning is identical across all settings.

This has practical relevance: it supports moving from a checkbox screening item (“was this pregnancy planned?”) to a situated, dialogic inquiry that surfaces the conditions under which a woman has come to orient herself toward, or away from, a future pregnancy. Such an approach is consistent with person-centered models of care and with shared decision-making, in which women’s own priorities guide the reproductive conversation [47].

The analysis both converges with and extends existing conceptual work, and it is instructive to contrast it with adjacent analyses rather than simply endorse them. The attributes identified in this analysis are consistent with theories grounded in planned behavior [48], which position intention as a proximal link between attitudes and action. However, these models do not explicitly account for how women appraise their personal, relational, socioeconomic, and health-related circumstances when considering their readiness for a future pregnancy. The present analysis extends this perspective by identifying contextual appraisal of readiness as a defining attribute and by incorporating the relational and structural dimensions highlighted in qualitative evidence, including gender inequality, socioeconomic constraints, and women’s multiple social roles in shaping reproductive agency [8,9,36]. These antecedents may be unequally distributed across social groups; in some contexts, structural inequities in education, employment, and living conditions appear to shape the circumstances under which women form pregnancy intentions [8,9,11].

This distinction is particularly relevant when contrasting pregnancy intention with the conceptualization proposed by Morin et al. [17], which describes pregnancy planning primarily in terms of preparatory actions undertaken before conception, such as modifying health behaviors or taking steps to prepare for pregnancy [17]. In contrast, the present analysis conceptualizes pregnancy intention as a prospective disposition that may exist without immediate preparatory action. A woman may consciously orient herself toward a future pregnancy and deliberate about her readiness without yet engaging in specific preparatory behaviors [8,9,36]. Thus, preparatory actions are better understood as possible expressions of pregnancy intention rather than as an essential component of the concept.

These distinctions carry implications for practice. Women who have not yet formed a specified pregnancy intention may nonetheless be engaged in a broader process of life planning, weighing educational trajectories, professional goals, relational stability, and personal circumstances in ways that shape, postpone, or condition future reproductive decisions [6,44]. For these women, support oriented toward life-course planning, rather than isolated reproductive screening, offers a more contextually appropriate response. Women who have formed a clear pregnancy intention but lack access to appropriate preconception support may face barriers to preparing for pregnancy in accordance with their health needs and personal circumstances [45].

The temporal attribute is particularly relevant to preconception care. Women who specify a horizon for the intended conception are more likely to initiate preparatory health behaviors and to engage with preconception services [30]. Clinically, this perspective supports moving beyond binary questions about whether a pregnancy is planned toward a person-centred assessment of how women envision a future pregnancy, including their readiness and the circumstances they consider relevant to conception. An equity-informed assessment also attends to the social determinants of health that shape each woman’s circumstances, helping professionals recognize and respond to disparities rather than attributing intention solely to individual choice [9,11]. Such an approach allows professionals to explore pregnancy intention prospectively without imposing a fixed temporal interval, while recognizing that the timeframe may vary according to each woman’s circumstances and reproductive plans [48].

Finally, the terminological instability documented in the literature has direct methodological and clinical consequences. Because pregnancy intention, intention to conceive, pregnancy desire, and pregnancy planning are used interchangeably, their conflation introduces systematic measurement error that impedes the accumulation of knowledge. The conceptual definition proposed here addresses this gap by offering a single referent that integrates prospective temporal orientation, a deliberative cognitive-affective process, and contextual appraisal of readiness, from which future measurement instruments and terminological standardization can be coherently derived.

## 6. Limitations

Several limitations should be acknowledged. The literature search was restricted to six databases and publications in English, Spanish, and Portuguese; although these sources were selected to capture the core international and Latin American literature relevant to pregnancy intention, relevant evidence indexed in other databases or published in other languages may not have been captured. The use of NOT operators, adopted to maintain the analytical focus on pregnancy intention in adult women, may have excluded records that contained these terms in ways that could have been compatible with the eligibility criteria. In addition, related terms such as pregnancy planning and pregnancy desire were not searched as independent concepts because an important aim of the analysis was to examine their conceptual relationship with pregnancy intention, given that these terms have been used interchangeably or with overlapping meanings in the literature. Rather than assuming equivalence, their use and relationship to pregnancy intention were examined in the retrieved literature to help clarify conceptual boundaries. Consequently, relevant sources using these terms without explicitly referring to pregnancy intention may not have been captured. Finally, the limited availability of prospective studies constrains the empirical evidence through which some attributes, particularly those related to temporal orientation, can be further characterized.

These limitations should be considered in light of the purpose and methodological nature of concept analysis, which is to clarify the meaning, defining attributes, and boundaries of a concept rather than to provide an exhaustive synthesis of the literature. As concept analysis is inherently interpretive and provisional, the proposed attributes and operational definition should be subject to further empirical validation and refinement across diverse populations and practice contexts.

## 7. Conclusions

Clarifying the concept of pregnancy intention is relevant to both nursing and reproductive health care. Demographic change, the postponement of motherhood, and the growing complexity of women’s reproductive trajectories require approaches that recognize the contextual and dynamic nature of reproductive intentions. Consistent with the critical literature on pregnancy intendedness [16], binary typologies that reduce pregnancy intention to a yes/no historical classification may not adequately capture the complexity of women’s reproductive experiences. Attending to pregnancy intention in an equity-informed, person-centred way can help reduce disparities in women’s health by recognizing how social determinants of health shape women’s health trajectories [7,9,11].

The three defining attributes identified—temporal orientation toward pregnancy, conscious deliberation about conceiving, and contextual appraisal of readiness—provide a conceptual structure for understanding pregnancy intention. The proposed definition distinguishes pregnancy intention from related concepts such as pregnancy planning and preconception and may provide a basis for future assessment approaches and prospective measurement.

## Figures and Tables

**Figure 1:**
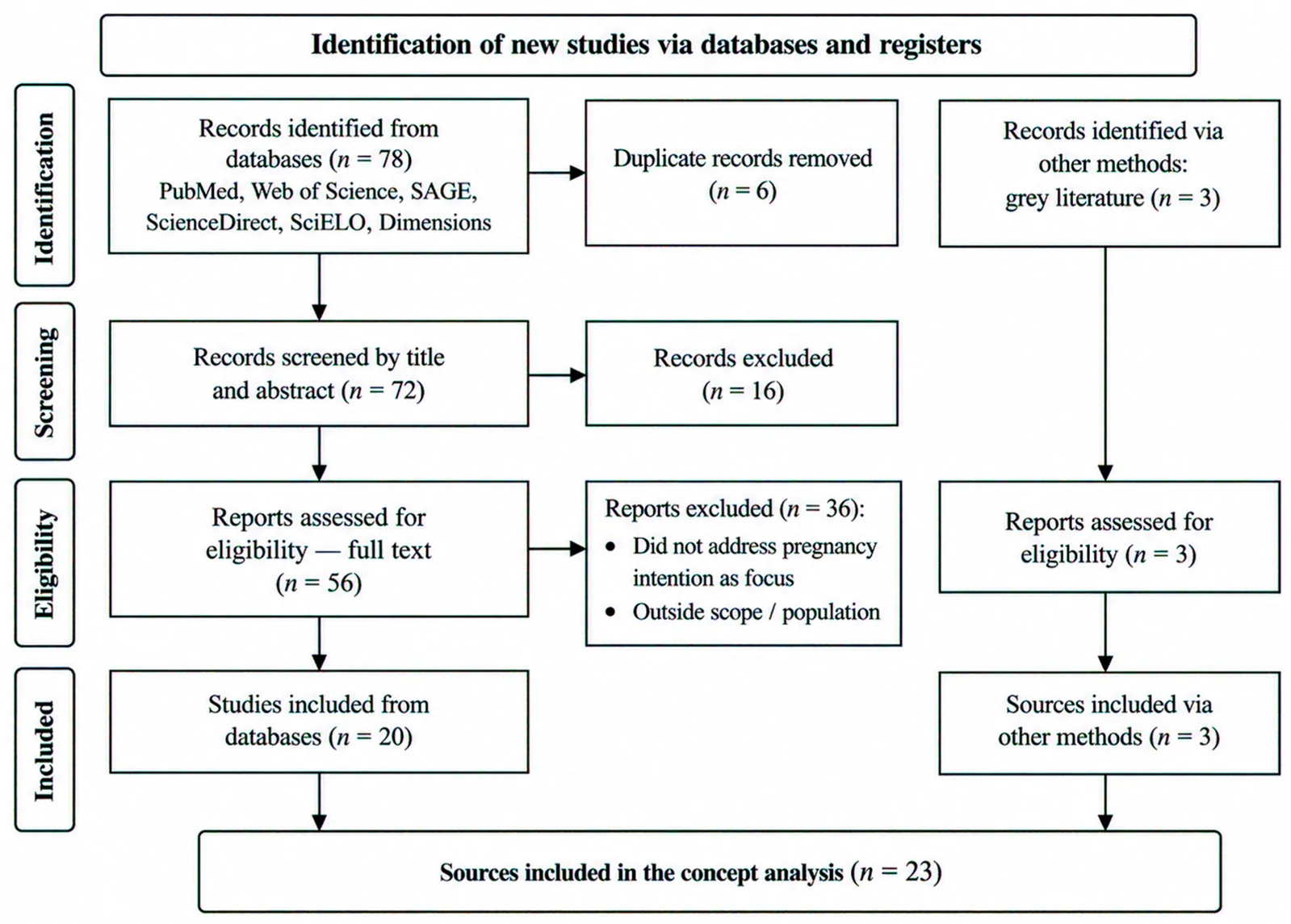
Study selection flow diagram (adapted from PRISMA 2020).

**Table 1:** Characteristics of sources included in the concept analysis.

Source [Ref]	Year	Country/Setting	Language	Publication/Design/Source Type
Bloch et al. [13] *	2012	USA	English	Quantitative (cohort)
Schytt [15]	2014	Sweden	English	Quantitative
Borrero et al. [8]	2015	USA	English	Qualitative
Lindberg et al. [29]	2015	USA	English	Quantitative (longitudinal)
Holliday et al. [9]	2018	USA	English	Qualitative
Lang et al. [30]	2018	Australia	English	Review
Hill et al. [31]	2019	Australia	English	Systematic mapping review
Khan et al. [10]	2020	Bangladesh	English	Quantitative
Maganha e Melo et al. [32]	2020	Brazil	Portuguese	Quantitative
Brunet et al. [33]	2020	Uruguay	Spanish	Quantitative
Kahn et al. [34]	2021	USA	English	Quantitative
Manze et al. [35]	2021	USA	English	Quantitative/mixed
Robbins et al. [36]	2021	USA	English	Quantitative
Mark & Cowan [37]	2022	USA	English	Quantitative
Schoenaker et al. [38]	2022	Australia	English	Quantitative
Sayler et al. [39]	2022	USA	English	Quantitative (dyadic)
Srinivasulu et al. [40]	2022	USA	English	Qualitative
Yu et al. [14]	2022	China	English	Systematic review/meta-analysis
Cha & Weitzman [11]	2023	USA	English	Quantitative
Temmesen et al. [41]	2023	Denmark	English	Qualitative meta-synthesis
Gawel [26]	2021	USA and Canada	English	Grey literature (professional news report)
Planned Parenthood [27]	2018	USA	English	Grey literature (institutional report)
United Healthcare [28]	2020	USA	English	Grey literature (institutional report)

Source: Prepared by the authors based on data extracted from the 23 studies included in the concept analysis. Note: Publication/design/source type describes the methodological or source characteristics of each publication and was not used as a criterion for determining the defining attributes. Conceptual contributions were extracted from the content of each source regardless of study design or publication type. The table includes the 23 sources retained for the concept analysis; other references cited in the manuscript were used as background or supporting literature. * Foundational publication published before the defined search period (2014–2025), retained because of its contribution to the conceptual development of pregnancy intention.

**Table 2:** Defining attributes of pregnancy intention and their relevance to nursing and reproductive health care.

Defining Attribute	Operational Description	Relevance to Nursing/Reproductive Health Care
Temporal orientation toward a future pregnancy	Prospective orientation toward a future pregnancy, with specific timeframes varying across measurement approaches rather than constituting a fixed conceptual boundary.	Supports assessment of whether and when women envision a future pregnancy and provides a basis for prospective reproductive health assessment and counselling.
Conscious deliberation about conceiving	An active cognitive and affective process that integrates desire, emotional readiness, partner agreement (where relevant), and perceived personal capacity, and that encompasses decisions about sexual activity and its timing.	Supports shared decision-making, fertility counseling, and woman-centered reproductive assessment.
Contextual appraisal of readiness	The woman’s appraisal of her readiness for a future pregnancy considering relevant personal, relational, socioeconomic, and health-related circumstances. This appraisal is defining, while the circumstances considered and their relative importance vary across women and contexts.	Guides a holistic, woman-centered assessment and enables tailored health promotion across the reproductive continuum.

Source: Prepared by the authors based on data extracted from the 23 studies included in the concept analysis.

**Table 3:** Mapping of sources to attributes.

Defining Attribute	Sub-Dimension	Sources Supporting the Identified Dimension
Temporal orientation toward a future pregnancy	Prospective orientation toward a future pregnancy	[13,26,27,37,39,41]
Conscious deliberation about conceiving	Cognitive-affective appraisal of readiness	[14,27,28,31,32,33]
Contextual appraisal of readiness	Health status	[34,37]
	Support network	[10,35,40]
	Socioeconomic conditions	[11,32,34,37]
	Women’s social roles and responsibilities	[8,37,41]

Source: Prepared by the authors based on data extracted from the 23 studies included in the concept analysis. Note: some sources informed more than one attribute.

## Data Availability

No new data were created or analyzed in this study. The data supporting the findings were derived from the 23 published sources included in the concept analysis and are available in the references cited in the manuscript.

## Supplementary Materials

The following supporting information can be downloaded at: https://www.mdpi.com/article/10.3390/nursrep16100351/s1, Supplementary Table S1. Database-Specific Search Strategies for the Concept Analysis of Pregnancy Intention. Supplementary Table S2. Bibliographic and Analytic Characteristics of the 23 Sources Included in the Concept Analysis of Pregnancy Intention.

## Public Involvement Statement

No public involvement in any aspect of this research.

## Guidelines and Standards Statement

This concept analysis followed Walker and Avant’s methodology [25]. PRISMA 2020 was used solely as a reporting aid to enhance transparency in the literature search and study-selection process.
