# Supplementary material for: Pregnancy Intention: A Concept Analysis
Source: Nurs Rep. 2026 Sep 30;16(10):351. doi: 10.3390/nursrep16100351 (PMC13708659; doi:10.3390/nursrep16100351)
Supplement: Supplementary file 1 [file nursrep-16-00351-s001.zip › nursrep-4531987-supplementary.pdf]

Supplementary Material S1

Supplementary Table S1. Database-Specific Search Strategies for the Concept Analysis of Pregnancy Intention

Manuscript: Pregnancy Intention: A Concept Analysis

The literature search covered publications from 2014 to 2025. The search was initiated in 2024 and updated in 2025. Search limits were English, Spanish, and Portuguese.

Database	Database-specific search strategy	Fields	Filters
PubMed	("pregnancy intention"[Title] OR "pregnancy intentions"[Title] OR "intended pregnancy"[Title]) AND (concept*[Title/Abstract] OR conceptual*[Title/Abstract] OR defin*[Title/Abstract] OR meaning*[Title/Abstract] OR construct*[Title/Abstract] OR framework*[Title/Abstract] OR "concept analysis"[Title/Abstract]) NOT (adolescent*[Title/Abstract] OR teenage*[Title/Abstract] OR surrogacy [Title/Abstract] OR "gestational surrogacy"[Title/Abstract])	Title; Title/Abstract	2014–2025; English, Spanish, Portuguese
Web of Science	TI= ("pregnancy intention" OR "pregnancy intentions" OR "intended pregnancy") AND TS=(concept* OR conceptual* OR defin* OR meaning* OR construct* OR framework* OR "concept analysis") NOT TS=(adolescent* OR teenage* OR surrogacy OR "gestational surrogacy")	TI; TS	2014–2025; English, Spanish, Portuguese
SAGE Journals	("pregnancy intention" OR "pregnancy intentions" OR "intended pregnancy") AND (concept* OR conceptual* OR defin* OR meaning* OR construct* OR framework* OR "concept analysis") NOT (adolescent* OR teenage*	Search interface fields	2014–2025; English, Spanish, Portuguese

	OR surrogacy OR "gestational surrogacy")		
ScienceDirect	TITLE-ABSTR-KEY ("pregnancy intention" OR "pregnancy intentions" OR "intended pregnancy") AND TITLE-ABSTR-KEY(concept* OR conceptual* OR defin* OR meaning* OR construct* OR framework* OR "concept analysis") AND NOT TITLE-ABSTR-KEY(adolescent* OR teenage* OR surrogacy OR "gestational surrogacy")	Title, abstract, keywords	2014–2025; English, Spanish, Portuguese
SciELO	("pregnancy intention" OR "pregnancy intentions" OR "intended pregnancy") AND (concept* OR conceptual* OR defin* OR meaning* OR construct* OR framework* OR "concept analysis") NOT (adolescent* OR teenage* OR surrogacy OR "gestational surrogacy")	Search interface fields	2014–2025; English, Spanish, Portuguese
Dimensions	"Pregnancy intention" OR "pregnancy intentions" OR "intended pregnancy" AND (concept* OR conceptual* OR defin* OR meaning* OR construct* OR framework* OR "concept analysis") NOT (adolescent* OR teenage* OR surrogacy OR "gestational surrogacy")	Search interface fields	2014–2025; English, Spanish, Portuguese

DATABASE	INITIAL SEARCH	UPDATE
**PUBMED**	April 2024	January 2025
**WEB OF SCIENCE**	May 2024	February 2025
**SAGE JOURNALS**	May 2024	March 2025
**SCIENCEDIRECT**	June 2024	April 2025
**SCIELO**	June 2024	May 2025
**DIMENSIONS**	July 2024	June 2025

Supplementary Table S2. Bibliographic and Analytic Characteristics of the 23 Sources Included in the Concept Analysis of Pregnancy Intention

Pregnancy Intention: A Concept Analysis

This matrix documents the bibliographic and analytic information extracted from the 23 sources presented in Table 1. The analytic fields reflect the conceptual synthesis reported in the manuscript. Where a source-specific element was not separately mapped in the manuscript, this is indicated rather than inferred.

Author / source [ref]	Year	Country / setting	Language	Publication type	Concept uses	Defining attributes	Antecedents	Consequences	Empirical referents
Bloch et al. [13]*	2012	USA	English	Quantitative (cohort)	Pregnancy intention; timing/desirability; contraceptive use	Temporal orientation toward a future pregnancy	Reproductive capability; prospective non-pregnant state	Preconception engagement; health-related behaviors	Timing/desirability questions; timing-preference items
Yu et al. [14]	2022	China	English	Systematic review / meta-analysis	Pregnancy intention in relation to smoking/alcohol use	Conscious deliberation about conceiving	Not individually specified	Health-related behaviors/outcomes	Not individually specified
Borrero et al. [8]	2015	USA	English	Qualitative	Pregnancy intention and planning	Conscious deliberation about conceiving; contextual appraisal of readiness appraisal of readiness (women's social roles and responsibilities)	Reproductive autonomy and agency; awareness of reproduction	Not individually specified	Qualitative accounts of intention/planning
Schytt [15]	2014	Sweden	English	Quantitative	Reproductive intentions and couple agreement	Conscious deliberation about conceiving; contextual appraisal of readiness	Reproductive autonomy and agency	Not individually specified	Assessment of reproductive intentions

						(relational context)			
Lindberg et al. [29]	2015	USA	English	Quantitative (longitudinal)	Pregnancy intentions and maternal/child health	Temporal orientation toward a future pregnancy	Not individually specified	Maternal and child health outcomes	Longitudinal assessment
Holliday et al. [9]	2018	USA	English	Qualitative	Pregnancy intention, reproductive coercion, partner violence	Contextual appraisal of readiness; reproductive agency	Reproductive autonomy and agency	Affective/contextual outcomes	Qualitative assessment
Lang et al. [30]	2018	Australia	English	Review	Preconception health in women of reproductive age	Contextual appraisal of readiness	Awareness of reproduction; reproductive capability	Preconception engagement	Not individually specified
Hill et al. [31]	2019	Australia	English	Systematic mapping review	Pregnancy intentions, lifestyle behaviours, psychological wellbeing	Conscious deliberation about conceiving; contextual appraisal of readiness	Not individually specified	Health behaviours; psychological wellbeing	Not individually specified
Khan et al. [10]	2020	Bangladesh	English	Quantitative	Pregnancy intention and maternal healthcare use	Contextual appraisal of readiness (support network)	Reproductive capability	Earlier/more sustained service engagement	Assessment at conception
Maganhae Melo et al. [32]	2020	Brazil	Portuguese	Quantitative	Contraceptive use and intention to become pregnant	Conscious deliberation about conceiving; contextual appraisal of readiness	Reproductive autonomy and agency; awareness of reproduction	Contraceptive/preconception behaviors	Assessment of intention to become pregnant
Brunet et al. [33]	2020	Uruguay	Spanish	Quantitative	Pregnancy intention and prenatal care	Conscious deliberation	Reproductive capability	Prenatal care engagement; possible risk reduction	Timing/desirability questions

						about conceiving			
Kahn et al. [34]	2021	USA	English	Quantitative	Changes in pregnancy intention following COVID-19	Contextual appraisal of readiness (health and socioeconomic conditions)	Reproductive autonomy and agency	Changes in pregnancy intention	Assessment of changes in intention
Manze et al. [35]	2021	USA	English	Quantitative / mixed	Pregnancy control, emotions, beliefs, pregnancy desires	Conscious deliberation about conceiving; contextual appraisal of readiness (support network)	Reproductive autonomy and agency	Affective outcomes	Assessment of control/emotions/beliefs/desires
Robbins et al. [36]	2021	USA	English	Quantitative	Pregnancy intention and maternal behaviors/experiences	Contextual appraisal of readiness; reproductive agency	Reproductive autonomy and agency	Maternal behaviors and experiences	Assessment of pregnancy intention
Mark & Cowan [37]	2022	USA	English	Quantitative	Pregnancy intentions and maternal outcomes	Temporal orientation toward a future pregnancy; contextual appraisal of readiness (women's social roles and responsibilities)	Reproductive capability	Maternal outcomes; life-planning implications	Timing-preference items
Schoenaker et al. [38]	2022	Australia	English	Quantitative	Pregnancy intention and psychological distress	Contextual appraisal of readiness	Contextual conditions	Psychological distress	Assessment of pregnancy intention

Sayler et al. [39]	2022	USA	English	Quantitative (dyadic)	Antecedents of pregnancy intention; prenatal father engagement	Temporal orientation toward a future pregnancy; contextual appraisal of readiness (relational context)	Reproductive autonomy and agency	Prenatal engagement	Dyadic/typological assessment
Srinivasulu et al. [40]	2022	USA	English	Qualitative	Women's perspectives on reproductive health screening	Contextual appraisal of readiness (support network)	Reproductive autonomy and agency	Not individually specified	Qualitative perspectives on screening
Gawel, R. [26]	2021	Not specified	English	Grey literature (professional news report)	Pregnancy intentions among nurses; fluidity of intentions	Temporal orientation toward a future pregnancy	Reproductive autonomy and agency	Changes or shifts in pregnancy intention over time	Reported assessment of pregnancy intentions
Cha & Weitzman [11]	2023	USA	English	Quantitative	Socioeconomic disparities in early pregnancy rates	Contextual appraisal of readiness (socioeconomic conditions)	Reproductive autonomy and agency	Pregnancy-related outcomes	Not individually specified
Temmesen et al. [41]	2023	Denmark	English	Qualitative meta-synthesis	Women's reflections on timing of motherhood	Temporal orientation toward a future pregnancy; conscious deliberation about conceiving; contextual appraisal of readiness (women’s social roles and	Reproductive autonomy and agency	Affective/life-planning considerations	Qualitative synthesis

						responsibilities)			
Planned Parenthood [27]	2018	USA	English	Grey literature (institutional report)	Pregnancy intention screening/provider assessment	Temporal orientation toward a future pregnancy; conscious deliberation about conceiving	Awareness of reproduction	Preconception engagement	Screening questions
United Healthcare [28]	2020	USA	English	Grey literature (institutional report)	Pregnancy intention screening/family planning	Conscious deliberation about conceiving; contextual appraisal of readiness	Awareness of reproduction	Preconception engagement	Screening questions

Note. *Bloch et al. [13] is a foundational publication published before 2014 and retained because of its contribution to the conceptual development of pregnancy intention. Gawel [26] was identified through grey-literature researching and retained as a professional news source reporting findings relevant to the fluidity and temporal dimension of pregnancy intentions. Some sources informed more than one conceptual dimension.

Publication type is reported as a descriptive characteristic of each source and was not used to determine conceptual relevance. Conceptual elements were extracted from the content of each source and subsequently mapped to defining attributes, antecedents, consequences, and empirical referents according to the predefined analytical criteria described in the Methods. Where a source contributed to more than one conceptual dimension, each contribution was recorded accordingly.
